# High Neuraxial Block in Obstetrics: A 2.5-Year Nationwide Surveillance Approach in the Netherlands

**DOI:** 10.1213/ANE.0000000000006866

**Published:** 2024-01-31

**Authors:** Ingrid C. M. Beenakkers, Timme P. Schaap, Oscar F. C. van den Bosch

**Affiliations:** From the * Department of Anesthesiology, Wilhelmina Children’s Hospital, University Medical Center Utrecht, Utrecht, the Netherlands; † Department of Obstetrics, Wilhelmina’s Children Hospital, University Medical Center Utrecht, Utrecht, the Netherlands.

## Abstract

**BACKGROUND:**

High neuraxial block is a rare but serious adverse event in obstetric anesthesia that can ultimately lead to respiratory insufficiency and cardiac arrest. Previous reports on its incidence are limited to populations in the United Kingdom and the United States. Little is known about the incidence and clinical features of high neuraxial block in the Netherlands, where the presence of anesthesiologists in the labor and delivery unit is comparatively lower. We aimed to assess the incidence and clinical features of high neuraxial block in obstetrics and to formulate ways to improve obstetric anesthesia on a national level.

**METHODS:**

This nationwide, prospective, population-based cohort study was designed to identify cases of high neuraxial block requiring ventilatory support (with supraglottic airway device or tracheal intubation) or cardiopulmonary resuscitation between November 2019 and May 2022. Cases were prospectively collected using the Netherlands Obstetric Surveillance System (NethOSS) in all hospitals with a maternity unit. Complete case file copies were obtained to determine risk factors and clinical course.

**RESULTS:**

During the study period, 5 cases of high neuraxial block requiring tracheal intubation were identified. The estimated incidence of high neuraxial block requiring tracheal intubation was 1 in 29,770 neuraxial procedures in labor (95% confidence interval, 1:12,758–1:91,659). Three of 5 identified cases occurred in the operating room after single-shot spinal anesthesia for Cesarean delivery after epidural analgesia in labor. One case developed in the labor ward due to an inadvertent intrathecal or subdural catheter placed for labor analgesia. The fifth case followed single-shot spinal anesthesia for elective Cesarean delivery. All 5 patients were successfully extubated in the operating room after Cesarean delivery, without the need for intensive care admission. There were no cardiac arrests and no neonatal deaths.

**CONCLUSIONS:**

High neuraxial block requiring tracheal intubation is a rare but impactful complication in obstetric anesthesia, potentially affecting both mother and fetus. Spinal anesthesia after epidural analgesia in labor is a common cause of high neuraxial block. Meticulous follow-up of epidurals in labor facilitates conversion to surgical anesthesia and may therefore reduce the need for spinal anesthesia after epidural analgesia. Large-scale surveillance systems in obstetric anesthesia are needed to identify those at risk, as well as to formulate further strategies to mitigate this burden.

**KEY POINTS**

- **Question:** What are the incidence, risk factors, and clinical features of high neuraxial block requiring ventilatory support (ie, supraglottic airway device or tracheal intubation) or cardiopulmonary resuscitation during labor in the Netherlands?
- **Findings:** In a 2.5-year national surveillance approach, the incidence was low (up to 1:12,758) and 3 of 5 cases occurred after single-shot spinal anesthesia for Cesarean delivery after epidural analgesia for labor.
- **Meaning:** Nationwide audits for monitoring adverse events in (obstetric) anesthesia are recommended to evaluate the quality of care in specific health care settings.

**See Article, page 1129**

High neuraxial block is the most frequent serious complication in obstetric anesthesia.^1^ It is characterized by excessive cephalad spread of local anesthetics, either through the intrathecal or epidural route. This leads to an uncomfortable sensation of tingling fingers, shortness of breath, and may eventually result in respiratory insufficiency, apnea, and ultimately in cardiac arrest.^2,3^ Depending on definition and population, the incidence of high neuraxial block in obstetrics ranges from 1:2971 to 1:16,200 neuraxial procedures.^1,4,5^ Risk factors include short body height, obesity, spinal deformities, and a spinal technique after failed conversion of labor epidural analgesia to cesarean delivery anesthesia.^1^

Compared to other countries in the developed world, the obstetric care system in the Netherlands is characterized by a low incidence of neuraxial analgesia for labor (23.6%) as well as a low rate of cesarean delivery (18.1%).^6^ Previous reports on the incidence of high neuraxial block are predominantly from health care settings where anesthetic interventions in obstetrics are more frequent.^1,4,5,7^ For instance, up to 80% of laboring women in the United States receive epidural analgesia.^8^ Little is known about the incidence of high neuraxial block in a population where neuraxial procedures are less common. Additionally, in current Dutch obstetric practice, anesthesiologists interrupt their daily work in the operating room to place epidurals in labor, and they are not typically part of the care team that is routinely present in the labor ward. These 2 distinct differences may have implications for the occurrence and characteristics of complications with regard to obstetric anesthesia.

In this study, we aim to describe the incidence and clinical features of high neuraxial block requiring ventilatory support or cardiopulmonary resuscitation in a nationwide 2.5-year surveillance approach in the Netherlands, to formulate recommendations on a national level.

## METHODS

This prospective nationwide population-based cohort study was performed between November 1, 2019 and May 1, 2022. Cases were identified using the Netherlands Obstetric Surveillance System (NethOSS), which is part of the Dutch birth registry and is used by the Dutch Society of Obstetrics and Gynecology Audit Committee on Maternal Mortality and Morbidity. The aim of NethOSS is to develop recommendations for clinical care, based on analysis of cases of severe maternal morbidity, mortality, and rare diseases.

In this study, all hospitals in the Netherlands with a maternity unit (n = 74) were asked to report cases of high neuraxial block requiring ventilatory support with a supraglottic airway device or tracheal intubation or requiring cardiopulmonary resuscitation. Each hospital had a reporting obstetrician who was contacted monthly with a request to submit cases or to reply with “nothing to report” if they had no cases that month. The initial study period of 12 months was extended by another 18 months because of an unexpectedly low number of reported cases. For every reported case, information with regard to the patient’s age, parity, estimated due date, clinical course, and details on anesthetic management were provided.

To estimate the incidence of high neuraxial block, the total number of births and the total number of neuraxial procedures in obstetrics were extracted from the national birth registry, which contains population-based data of 99% of all pregnancies in the Netherlands.^6^ Registry data from July 1, 2019 until December 31, 2021 were used as this was the most recent 2.5-year period available at the time of this study. The total number of Cesarean deliveries under neuraxial anesthesia and the total number of vaginal deliveries with neuraxial analgesia were extracted to determine the number of deliveries that were exposed to a neuraxial anesthetic procedure. The Clopper-Pearson method was used to determine the 95% confidence interval for the incidence of high block requiring tracheal intubation.

The use of data from the NethOSS project for research purposes was centrally approved by the medical ethics committee of Leiden University Medical Center (P12-216/SH/sh, dated March 12, 2013). Informed consent was deemed not applicable for performing confidential enquiries with anonymized data.

## RESULTS

The estimated number of births in our 2.5-year study period was 426,483, of which 148,871 were exposed to a neuraxial procedure. During the 2.5-year study period, a high neuraxial block was reported in 7 cases. On detailed case review, 5 cases met the inclusion criteria of high neuraxial block and ventilatory support with tracheal intubation. In the 2 remaining cases, the high block was managed with supplemental oxygen via mask without tracheal intubation or bag mask ventilation, and they were therefore excluded. The estimated incidence of high neuraxial block requiring tracheal intubation was therefore 1 in 29,770 neuraxial procedures in labor (95% confidence interval, 1:12,758–1:91,659). The patient characteristics, anesthetic management, and outcomes are summarized in the Table. Body mass index ranged from 23 to 30 kg/m^2^ before pregnancy. All deliveries were at term.

**Table.** Cases of High Neuraxial Block Requiring Tracheal Intubation in A 2.5-Year Nationwide Audit in the Netherlands

Case	BMI	Anesthetic	Epidural procedure	Spinal procedure	Location^a^	Cardiac arrest	Apgar scores^b^
1	23	SSS for CD after LEA	bupi 0.1%/suf 0.2 µg/mL 10 mL/h, 2 × 4 mL bolus^c^	L?, arti 60 mg	OR	-	9/9^d^
2	29	SSS for CD after LEA	bupi 0.1%/suf 0.2 µg/mL 10 mL/h	L1–L2, HB 10 mg	OR	-	1/6/9 and 1/5/9^e^
3	29	SSS for CD after LEA	bupi 0.1%/suf 0.2 µg/mL 8 mL/h	L4–L5 HB 12.5 mg	OR	-	5/7
4	26	LEA	L1–L2, lido 40 mg bolus	N/A	LW	-	5/8
5	30	SSS for CD	N/A	L2–L3, HB 14 mg	OR	-	9/10

Abbreviations: arti, articaine; BMI, body mass index before pregnancy; bupi, bupivacaine; CD, Cesarean delivery; fent, fentanyl; HB, hyperbaric bupivacaine; LEA, labor epidural analgesia; lido, lidocaine; LW, labor ward; L?, unknown spinal interspace level; N/A, not applicable; OR, operating room; SSS, single-shot spinal; suf, sufentanil.

a Location where the high neuraxial block occurred.

b After 1/5 (/10) min.

c Last bolus 2 h before spinal anesthesia.

d Neonate admitted for respiratory support.

e Twin delivery; second neonate admitted for continuous positive airway pressure ventilation.

Four out of 5 cases (#1, #2, #3, and #4) were patients with an intended vaginal delivery, for whom epidural analgesia was administered. Three of those cases (#1, #2, and #3) underwent secondary cesarean delivery due to failure to progress in the first stage of labor. The anesthetic management for those patients consisted of single-shot spinal anesthesia with hyperbaric bupivacaine (n = 2; 10 mg and 12.5 mg) or with articaine (n = 1; 60 mg). There was no attempt to use the labor epidural catheter to facilitate surgical anesthesia in these 3 cases. The reason for single-shot spinal anesthesia instead of epidural top-up for cesarean delivery could be retrieved in 2 cases, and this was due to unsatisfactory analgesia for labor. These 3 patients developed a fast onset of respiratory insufficiency and apnea within 10 minutes of the intrathecal injection.

The other case (#4) with an intended vaginal delivery was characterized by an inadvertent intrathecal or subdural catheter. A test dose of lidocaine 40 mg was administered in the labor ward and resulted in severe hypotension, dysphagia, and respiratory insufficiency, for which the patient was transported to the operating room for emergency Cesarean delivery under general anesthesia with tracheal intubation.

The remaining case (#5) was a patient for a repeat Cesarean delivery, who underwent single-shot spinal anesthesia with hyperbaric bupivacaine 14 mg. This resulted in a fast onset of bradycardia and severe hypotension, followed by apnea. The patient was rapidly intubated and stabilized with intravenous fluids and vasopressors.

All patients were extubated shortly after cesarean delivery, with no need for intensive care admission. There were no cardiac arrests. Two patients were offered psychological support in the postpartum period to address ongoing anxiety and/or the development of posttraumatic stress disorder.

There were no neonatal deaths and Apgar scores are shown in the Table. Two neonates needed respiratory support in the neonatal ward.

## DISCUSSION

In this 2.5-year nationwide surveillance approach, high neuraxial block in labor requiring intubation in the Netherlands was a rare but impactful event, and direct intervention prevented further harm.

The low number of identified cases is in line with previous studies on high neuraxial block in obstetrics. Previous estimates regarding its incidence in the United Kingdom have ranged from 1 in 2971 to 1 in 16,200 neuraxial procedures.^4,5^ In the United States, the Serious Complication Repository Project was developed by the Society for Obstetric Anesthesia and Perinatology to establish the incidence of serious complications related to obstetric anesthesia. Data in that repository from more than 257,000 anesthetics showed a rate of high neuraxial block of 1 in 4336 anesthetics.^1^ To the best of our knowledge, we present the first study outside of the United States and the United Kingdom to shed light on this serious adverse event.

High neuraxial block could ultimately lead to cardiac arrest, although we identified no such cases. The United Kingdom Obstetric Surveillance system previously reported that 22% of cardiac arrests in pregnancy were caused by complications of a neuraxial block.^2^ Reassuringly, a similar surveillance study in the Netherlands reported that only 3% (n = 1) of cardiac arrests in pregnancy were related to anesthetics.^9^

In our study, single-shot spinal anesthesia was used after epidural analgesia for labor in 3 out of 5 cases. Despite an administered dose of hyperbaric bupivacaine lower than the ED-95 previously described for Cesarean delivery, it led to a high neuraxial block with respiratory insufficiency requiring intubation.^10–14^ This confirms that the effects of intrathecal local anesthetics with concurrent epidural analgesia in labor are unpredictable and that caution is warranted in this specific setting.

It is well established in the literature that the practice of spinal anesthesia after failed conversion of labor epidural analgesia to cesarean delivery anesthesia is a risk factor for the development of high neuraxial block.^1,15,16^ However, previous reports have suggested that spinal anesthesia after prolonged epidural analgesia without recent top-up appears safe.^17,18^ Our study reveals that single-shot spinal anesthesia after epidural analgesia can lead to severe complications even in the absence of a surgical top-up or recent epidural boluses.

Alternatively, a well-functioning epidural catheter for labor may be used to facilitate an intrapartum Cesarean delivery.^19–21^ This requires routine and regular assessment of labor epidural analgesia to optimize labor analgesia as well as to identify and replace those that will likely fail to convert to surgical anesthesia, in case a cesarean delivery is required.^22–24^ We will advocate further implementation of this recommendation on a national level to allow successful and timely top-up of epidural catheters and to further reduce the rate of high block in obstetrics.

We identified a low number of high neuraxial blocks in obstetrics. This may be explained by the country’s low rate of obesity, which is a known risk factor for high neuraxial block.^1,25,26^ Additionally, the dose of intrathecal bupivacaine used for Cesarean delivery in labor may impact the risk of high neuraxial block. A higher dose may trigger a high block, whereas a lower dose may lead to insufficient analgesia for Cesarean delivery. Further studies are needed to explore whether the low rate of high neuraxial block is accompanied by a high rate of insufficient analgesia for cesarean delivery on a national level.

It is important to highlight that an unexpected high block in obstetrics can be extremely frightening for the parturient and may be a cause of long-lasting psychological distress. In our cohort, 2 patients described a horrifying fear of dying. These patients were offered debriefing with the attending anesthesiologist as well as further support from a medical psychologist or social worker. We emphasize that adequate psychological support is warranted in the postpartum period to address ongoing anxiety and, potentially, to recognize and treat the development of a posttraumatic stress disorder.

The strength of this study is the prospective population-based study design with the participation of all Dutch hospitals with labor wards. The NethOSS registration system was used to remind reporting physicians on a monthly basis. An important limitation of this study is that recall bias may have led to under-reporting. Potentially, not all instances of high neuraxial block requiring tracheal intubation were noted by the reporting obstetrician. However, we emphasize that high neuraxial block requiring tracheal intubation is remarkable for all caregivers involved, and we therefore believe it is unlikely that a large number of cases were missed during the study period. Second, our study was largely during the coronavirus disease-2019 (COVID-19) pandemic and this may have impacted the management of respiratory insufficiency associated with high neuraxial block. Potentially, borderline cases of high neuraxial block were managed without airway manipulation whereas, under nonpandemic times, these would have been managed with tracheal intubation. Thirdly, we were limited by the information provided by the reporting physicians, and we were therefore unable to retrieve information regarding long-term psychological follow-up.

We conclude that high neuraxial block in labor is uncommon in the Netherlands and that it occurs predominantly after spinal anesthesia for Cesarean delivery, after epidural analgesia for labor. Meticulous management of neuraxial blocks in labor may further reduce the rate of high neuraxial block. We advocate that large-scale surveillance systems are warranted to assess rare adverse outcomes in (obstetric) anesthesia.

## ACKNOWLEDGEMENTS

We kindly thank Ageeth N. Rosman, PhD, for her help with acquiring data from the Dutch national birth registry (Perined).

## DISCLOSURES

**Name:** Ingrid C. M. Beenakkers, MD.

**Contribution:** This author helped in study conception and design, data collection, analysis and interpretation of results, and writing of the original draft.

**Name:** Timme P. Schaap, MD, PhD.

**Contribution:** This author helped in the study conception and design, analysis, and interpretation of results, and reviewing and editing of the work.

**Name:** Oscar F. C. van den Bosch, MD.

**Contribution:** This author helped in study design, analysis and interpretation of results, and reviewing and editing of the work.

**This manuscript was handled by:** Jill M. Mhyre, MD.
